# Classification of Whisker Deflections From Evoked Responses in the Somatosensory Barrel Cortex With Spiking Neural Networks

**DOI:** 10.3389/fnins.2022.838054

**Published:** 2022-04-14

**Authors:** Horst Petschenig, Marta Bisio, Marta Maschietto, Alessandro Leparulo, Robert Legenstein, Stefano Vassanelli

**Affiliations:** ^1^Faculty of Computer Science and Biomedical Engineering, Institute of Theoretical Computer Science, Graz University of Technology, Graz, Austria; ^2^NeuroChip Laboratory, Department of Biomedical Sciences, University of Padova, Padova, Italy

**Keywords:** biomarker, artificial intelligence, computing, spiking neural network, neural coding, neural decoding, cortical microcircuits, liquid state machine

## Abstract

Spike-based neuromorphic hardware has great potential for low-energy brain-machine interfaces, leading to a novel paradigm for neuroprosthetics where spiking neurons in silicon read out and control activity of brain circuits. Neuromorphic processors can receive rich information about brain activity from both spikes and local field potentials (LFPs) recorded by implanted neural probes. However, it was unclear whether spiking neural networks (SNNs) implemented on such devices can effectively process that information. Here, we demonstrate that SNNs can be trained to classify whisker deflections of different amplitudes from evoked responses in a single barrel of the rat somatosensory cortex. We show that the classification performance is comparable or even superior to state-of-the-art machine learning approaches. We find that SNNs are rather insensitive to recorded signal type: both multi-unit spiking activity and LFPs yield similar results, where LFPs from cortical layers III and IV seem better suited than those of deep layers. In addition, no hand-crafted features need to be extracted from the data—multi-unit activity can directly be fed into these networks and a simple event-encoding of LFPs is sufficient for good performance. Furthermore, we find that the performance of SNNs is insensitive to the network state—their performance is similar during UP and DOWN states.

## 1. Introduction

Brain-computer interfaces (BCI) typically rely on personal computers to process brain signals. In the perspective of neuroprosthetics or other neurological applications such as adaptive neuromodulation, miniaturization of the processing unit is crucial to the engineering of minimally invasive implantable devices. Neuromorphic (a.k.a. brain-inspired) computing architectures represent an interesting option which may be advantageous in terms of power efficiency and adaptability to intrinsically variable and noisy brain signals (Vassanelli and Mahmud, 2016; Osborn et al., 2018; Buccelli et al., 2019; George et al., 2020; Serb et al., 2020; Zeng et al., 2021). Among neuromorphic systems, artificial networks of spiking neurons share with biological neurons the spike-based language and synaptic processing rules (Mead, 2020). They inherit from their biological counterparts their energy-efficiency and inherent temporal processing capabilities (Davies et al., 2021). As such, in a long-term vision, they represent the ideal replacement or rehabilitation support for native brain circuits affected by neurological deficits. With invasive interfaces, brain signals are sensed intracranially and fall into two categories: local field potentials (LFPs) and extracellular spikes (Buzsáki et al., 2012). Both convey information on neural activity, but with different nuances, and both have been proved effective in the realm of neuroprosthetics and adaptive brain stimulation [e.g., to control a prosthetic limb (Leuthardt et al., 2006) or for adaptive DBS in Parkinson's patients (Priori et al., 2013)]. LFPs originate from neural populations and are commonly bandpass filtered with an upper cutoff frequency of about 300 Hz. Extracellular spikes are faster signals (typically between 300 and 3,000 Hz) and represent signatures of single neurons. When the sensing electrode collects multiple, overlapping spikes from a small neuronal population, the signal is named multi-unit-activity (i.e., MUA) in contrast to well-isolated spikes from single neurons that are called single-units. A crucial open question is whether spikes or LFPs are preferable for BCI-based applications. Extracellular spikes are easier to interpret given their single-neuron origin. Moreover, they are “binary” events that can be detected and sorted, which makes decoding more tractable (Quiroga and Panzeri, 2009). On the other hand, they may represent a minority of the neurons involved in a given processing task. In addition, extracellular spikes tend to fade away leading to signal loss in chronic implants, mainly because of gliosis that heavily affects single neuron-microelectrode interfacing (Szostak et al., 2017). LFPs are more robust over time in chronic conditions (Abosch et al., 2012) and may be preferable in clinic for long term implants (Jackson and Hall, 2017; Tekriwal et al., 2019). However, it is more difficult to relate them to the underlying neural activity and to devise decoding strategies (Buzsáki et al., 2012; Einevoll et al., 2013). Whether LFPs convey more or less information with respect to spikes remains debated. As a lumped representation of the neuronal population, the LFP signal is likely missing information from “key-player” single neurons, but it may capture a more complete picture reflecting collective processing of the network. In general, the scenario may change depending on the targeted brain structure and compromises may have to be found which vary from case to case. An additional fundamental aspect when dealing with evoked responses is their dependence on basal brain activity (Petersen et al., 2003), which may pose different challenges for LFPs or spikes. Solutions for online-processing spikes and LFPs that are or may become suitable for brain implants have been proposed based on analog and digital processors (Tambaro et al., 2021). Only a minority have explored neuromorphic architectures and a few of them spiking neural networks (SNNs) (Boi et al., 2016; Werner et al., 2016; Mukhopadhyay et al., 2021; Sharifshazileh et al., 2021).

In this work, we investigate the problem of brain signal classification with SNNs, focusing on evoked sensory responses in the rat barrel cortex. This system has the advantage of a one-to-one mapping of the sensory organ (the whisker) to a single cortical column (Diamond et al., 2008) which is experimentally accessible through an implanted shank-shaped multi-electrode array across cortical layers. Moreover, in the anesthetized animal, single whisker deflection can be operated by a piezoelectric device which allows for a tight control and fine tuning of the sensory input (Mariani et al., 2021). This experimental arrangement enabled us to investigate the capability of SNNs to classify whisker deflections of different amplitudes. We compared the classification performance when the network input was based on MUA and LFPs, and during UP and DOWN states of spontaneous brain activity. SNNs have been shown to achieve state-of-the art performance on a variety of temporal classification tasks (Bellec et al., 2018b; Salaj et al., 2021). However, these models were trained with complex learning algorithms that cannot readily be implemented in neuromorphic hardware. We therefore focused in this work on the liquid state machine (LSM) model (Maass et al., 2002), which is well-suited for neuromorphic implementation and achieves good performance on less demanding tasks (Verstraeten et al., 2005; Maass, 2011). We found that the LSM approach performs better or on par with various machine learning methods including artificial neural networks and SNNs fully trained with advanced training methods. Furthermore, our results indicate that no sophisticated feature extraction from the raw signals is necessary to achieve good performance. This suggests that classification of whisker stimulation intensities based on LFP signals as well as multi-unit activity (MUA) recorded in rat barrel cortex is viable using neuromorphic hardware.

## 2. Results

### 2.1. Experimental Protocol and Signal Preprocessing

**Figure 1:**
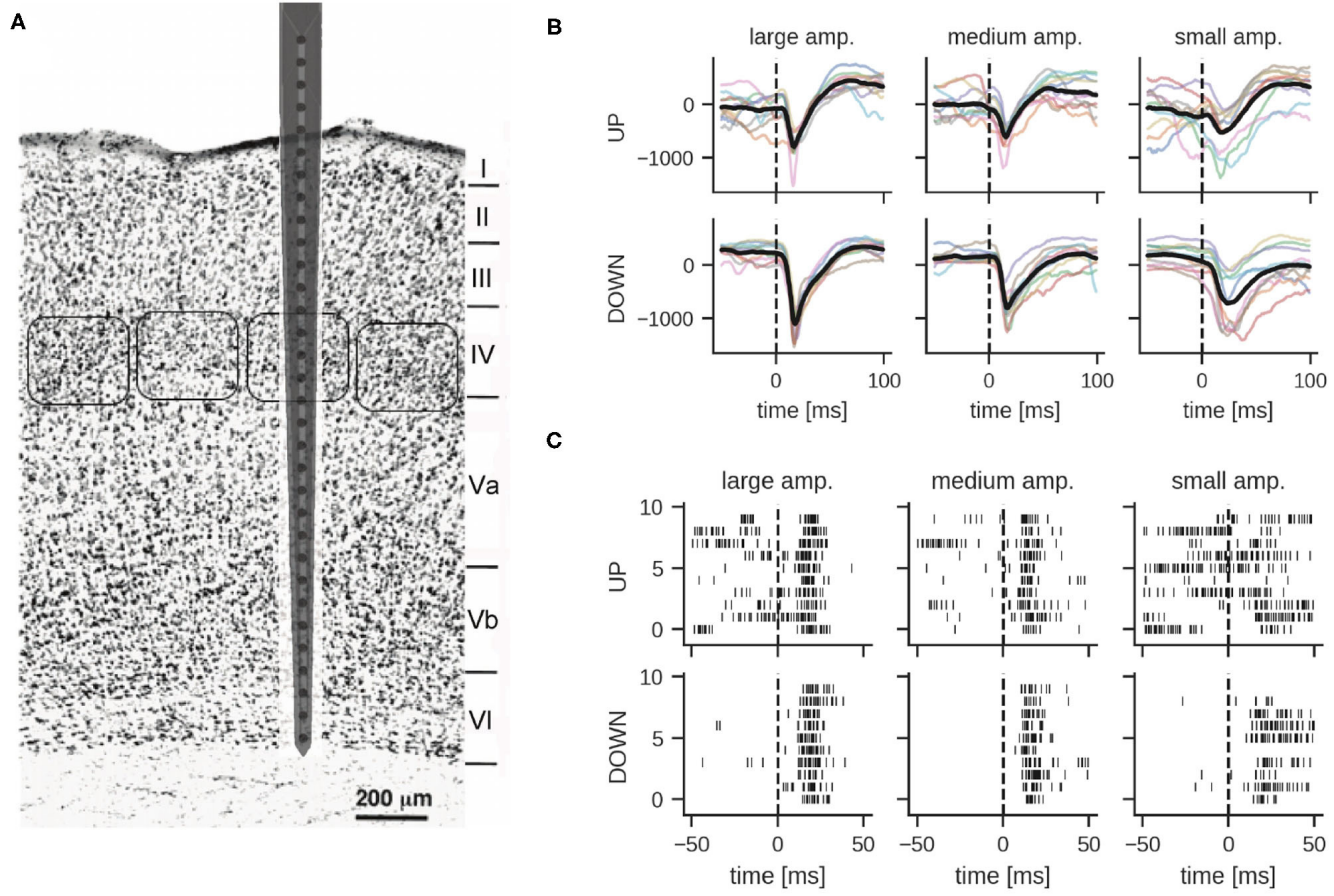
**(A)** Schematic representation of the neural probe with thirty-two recording microelectrodes implanted in the somatosensory barrel cortex. Twenty-seven out of thirty-two microelectrodes span the six cortical layers, while five recording sites are protruding within the grounded recording solution bathing the brain. Barrel fields at the level of the fourth layer are approximately indicated by rounded boxes. Adapted from Zhang and Deschênes (1997). Copyright 1997 Society for Neuroscience. **(B,C)** Examples of local field potentials and multi-unit activity in response to whisker stimulation at time 0 (10 trials shown). **(B)** Comparison of LFP signals from layer IV per stimulation intensity and global network state; different colors correspond to different stimulation trials (units in μV). The dashed line indicates stimulation onset, the solid thick line shows the mean LFP signal. **(C)** Multi-unit activity recorded from electrode 18 in layer IV. Each row shows the response to one stimulation of the given amplitude during UP or DOWN states.

Using a neural probe with 32 microelectrodes (27 of them spanning across the cortex), we recorded stimulus-evoked responses from the barrel cortex in terms of both LFPs and spikes, mostly within MUA events (see Figure 1A and Section 4 for details on animal preparation, surgical implantation, whisker stimulation and data acquisition). In brief, the whisker corresponding to a recorded barrel column was deflected using a closed-loop controlled piezoelectric bender (Mariani et al., 2021) by administering 5 ms voltage pulses with a 10 s inter-stimulus interval to avoid adaptation phenomena in the somatosensory neural pathway. Deflections of different amplitudes (large, medium and small) were delivered corresponding, respectively, to voltage pulses of 2.8, 2.0, and 1.6 V at the input of the piezoelectric control system. In the representative experiment reported below, the number of trials was 61 for the large, 63 for the medium, and 61 for the small amplitude stimulus. In total, there were 185 stimulation trials in the dataset. LFPs and spikes were measured in all six cortical layers of the single barrel simultaneously (see Figure 1A for a schematic of the neural probe inserted in the barrel column and spanning all cortical layers).

LFP signals were extracted by bandpass filtering raw traces between 0.1 and 300 Hz. Figure 1B illustrates examples of responses for the three different stimulation intensities. As reported previously (Civillico and Contreras, 2012), responses were conditioned by the current network state, i.e., by whether the stimulus was delivered during an epoch of high (UP) or low (DOWN) spontaneous activity. The traces show a clear response to the stimulus approximately 25 ms after whisker stimulation. DOWN states usually favored responses of larger amplitude, particularly for what concerns the negative peak (which is supposedly reflecting excitatory input currents to the cortical neurons), see also Section 2.2. Noteworthy, for the analysis reported in the following paragraphs we mainly focused on LFPs recorded in layer IV, unless stated otherwise. Indeed, this cortical input layer of the whisker somatosensory pathway is characterized by a strong response to sensory stimulation and classifiers trained on LFPs recorded from this layer performed best (see Section 2.3.4 for details). Importantly for our classification study, and in agreement with earlier studies (Petersen et al., 2003; Temereanca and Simons, 2003), the amplitude of the stimulus-evoked response clearly correlates with stimulation intensity, albeit with a significant variability across trials (Figure 1B).

In order to obtain spiking activity, the recorded signal was bandpass filtered in the frequency range between 300 and 3,000 Hz. Spiking activity in each of the 27 electrodes was detected by simple thresholding, but no spike-sorting was applied to the MUA data. As reported below, for our spiking classifiers, these spike events were directly provided as inputs to the networks. Figure 1C shows example MUA traces from one recording electrode in both UP and DOWN state conditions. Note the variability of the response in particular for stimulations during an UP state.

### 2.2. Statistical Analysis of Features Extracted From Evoked Responses

In order to understand what components of LFPs and MUAs carry relevant information about whisker stimulation intensity, we first performed a statistical analysis on features of the signals that were selected based on our own experimental experience and on previous work (Wang et al., 2018).

#### 2.2.1. Statistical Results on Features Extracted From Evoked LFPs

We extracted four characteristic features from the evoked LFPs. These features were the Negative Peak Amplitude (NPA), the Positive Rebound Amplitude (PRA), the Response Onset Latency (ROL), and the time-normalized LFP (tLFP). Our aim was to investigate from a statistical perspective whether and how basal activity affects stimulus-evoked responses, and whether different stimulation intensities elicit responses with distinguishable features.

**Figure 2:**
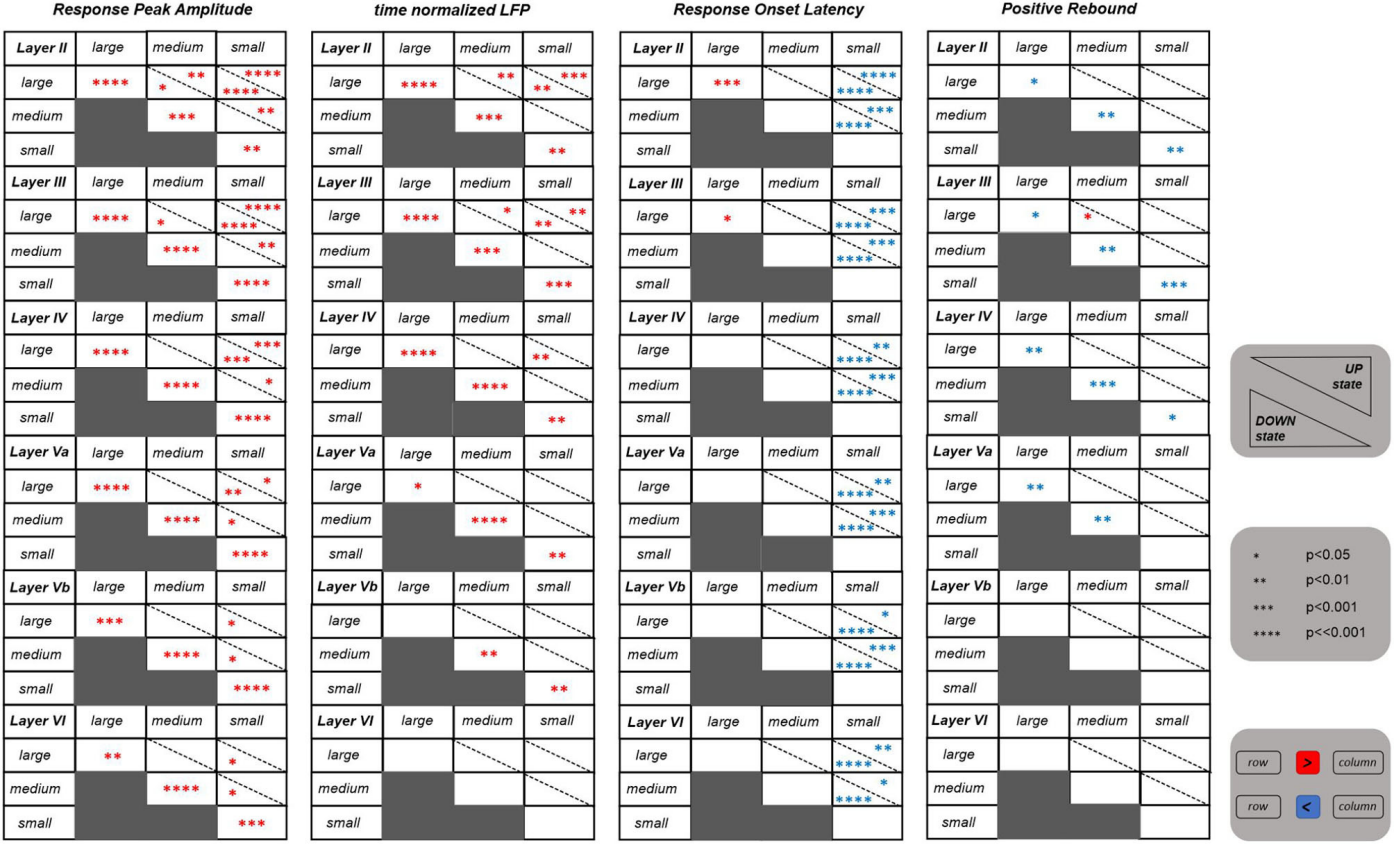
Statistical results for four features extracted from the evoked LFPs. Each table shows the results referring to one specific feature for all the six investigated layers. Each table is consisting of six blocks, each one corresponding to one layer as indicated on the top left of the block. The statistics was performed by comparing between the three different stimulation intensities (i.e., large, medium, and small) as well as between the UP and DOWN state condition. The number of asterisks indicate the strength of statistical significance (i.e., *, **, ***, **** correspond, respectively, to *p* ≤ 0.05, *p* ≤ 0.01, *p* ≤ 0.001, *p* ⋘ 0.001). The asterisks reported in cells of the main diagonal of each layer block compare the feature values between the UP and DOWN state condition for each stimulation intensity separately. The asterisks outside the main diagonal compare the values of each feature for the UP (upper right triangle) and DOWN state (lower left triangle) conditions separately, comparing between pairs of different stimulation intensities, at each layer. Red and blue asterisks indicate, respectively, a significant increase and decrease of values of a row against the corresponding column.

The tables shown in Figure 2 summarize the results obtained for all the above cited investigated features. We first asked whether the values of these features differ significantly between UP and DOWN states. In brief, we found significant differences for the *NPA* feature within all investigated layers, and, in particular, the responses' negative peak amplitude was significantly larger when the whisker stimulus was falling during the DOWN than during the UP state. Also the *tLFP* feature within the more superficial and middle layers (i.e., layers II, III, IV, and Va), was following a similar trend. Instead, the *PRA* feature in superficial and middle cortical layers (i.e., layers II, III, IV, and Va), was characterized by a larger amplitude during the UP state.

Concerning the three different stimulation intensities, strong differences were observed for the *NPA* feature within the more superficial layers (i.e., layers II, III), and this for both DOWN and, more weakly, UP states. In practice, in these superficial layers, the negative peak amplitudes were significantly different between all three levels of stimulus intensity, a trend that was “inherited” also by *tLFP*. In deeper layers, instead, the statistical difference was maintained only between large and small stimulus intensities, and vanishing in layer VI for *tLFP*. The responses' onset latency (*ROL*) was significantly higher after a small intensity stimulus compared to both a large and a medium stimulus, which is reflected in the table. The cortical state was influencing the latency, with the UP state delaying the response onset with respect to the DOWN state at small stimulus intensity and, on the contrary, anticipating it for large intensity stimulation. Finally, *PRA* was rather invariant across intensities. Intriguingly, features of layer IV, where the LFP response is typically larger, seemed to be slightly less informative compared to layers II and III, with respect to stimulus intensity discrimination.

Overall, these results suggest that selected LFP features, taken collectively across layers, carried sufficient information to discriminate between stimulus intensities and whether they were occurring during UP or DOWN states. Responses in superficial layers and, among features, the amplitude of the negative peak appeared to be the most effective.

#### 2.2.2. Statistical Analysis of Features Extracted From Evoked Spike Responses

We extracted three features from the evoked MUA (see Section 4 and **Figure 7A**). These features were: The evoked MUA latency, the evoked MUA duration, and the evoked MUA firing rate. As for the features extracted from evoked LFPs, the aim was to investigate whether and how basal activity (i.e., UP or DOWN state condition at stimulation occurrence) affects stimulus-evoked MUAs, and whether different stimulation intensities induce responses with clearly discernible features. Indeed, the stimulation protocol was very effective in always inducing a supra-threshold response, i.e., a single MUA observed within the first 100 ms from stimulation delivery. This aspect allowed to effectively investigate differences observed in the features extracted from evoked MUAs elicited after stimuli delivered during UP and DOWN states.

**Figure 3:**
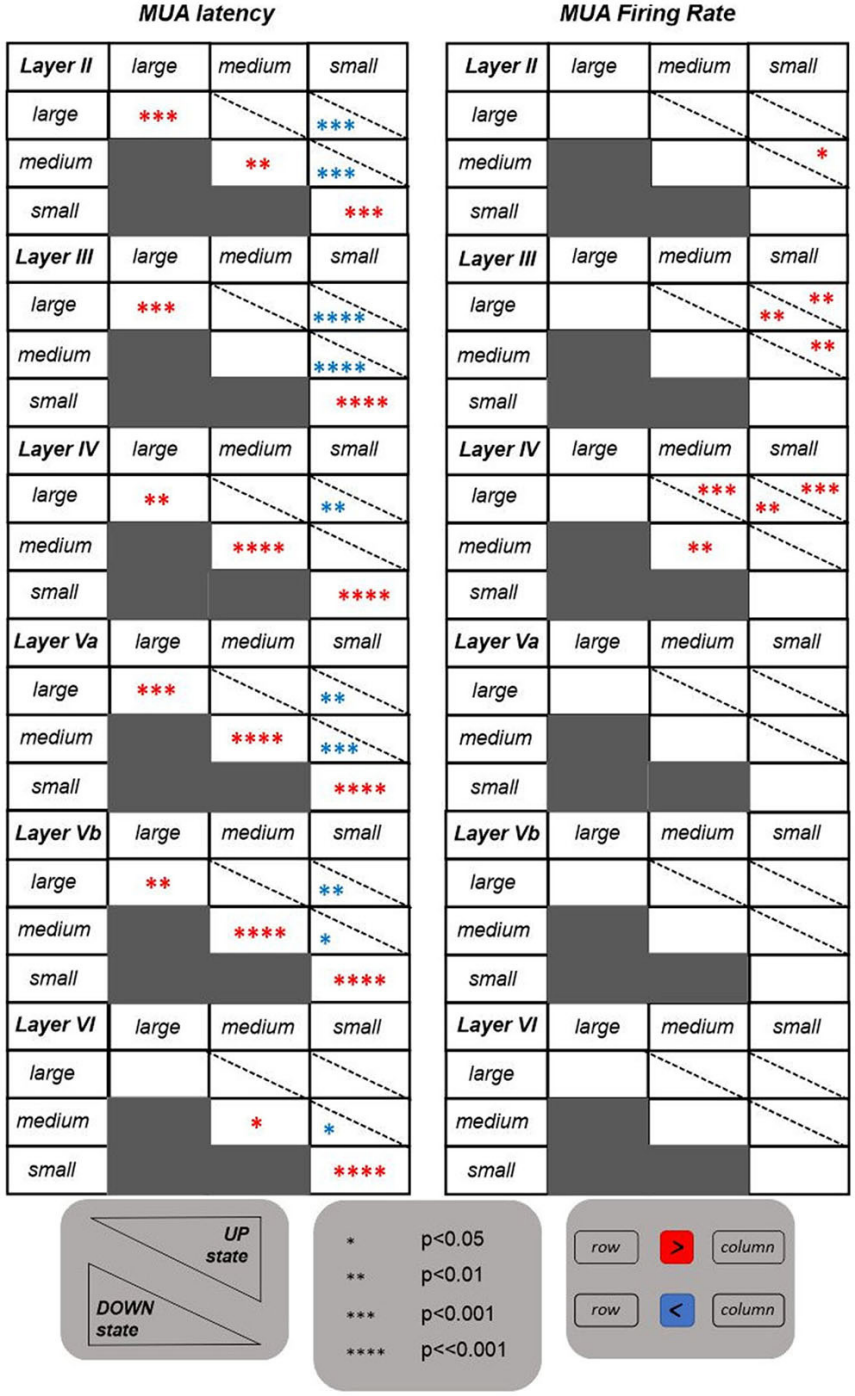
Tables reporting the statistical results for the features extracted from the evoked MUAs. Same as in Figure 2, but referring to the two features extracted from MUAs.

The tables shown in Figure 3 summarize the results obtained for the investigated features. The MUA duration is not included in this summary. It appeared to be not informative since no significant differences have emerged from the statistical analysis. Contrary to the results obtained for LFP features, the MUA latency is the only informative feature. Indeed, it is able to discriminate on the one hand between UP and DOWN state conditions, with latency values significantly higher for stimuli occurring in a DOWN state, and, on the other hand, between responses following higher intensity stimulations (i.e., large and medium) and responses following a low intensity one (i.e., small). In the latter case, the significant difference is only observed following stimuli delivered during a DOWN state condition, with response latency values being significantly higher after small intensity stimuli compared to both medium and large intensities, also in accordance with what was observed for the *ROL* feature. These trends were found across all investigated layers.

Regarding the MUA firing rate, instead, the only significant differences are observed within the middle layers (i.e., layers III and IV), with evoked MUA firing rate values significantly higher when stimulating with a large compared to a small intensity, in both UP and DOWN state conditions.

### 2.3. Stimulation Intensity Classification With Spiking Neural Networks

**Figure 4:**
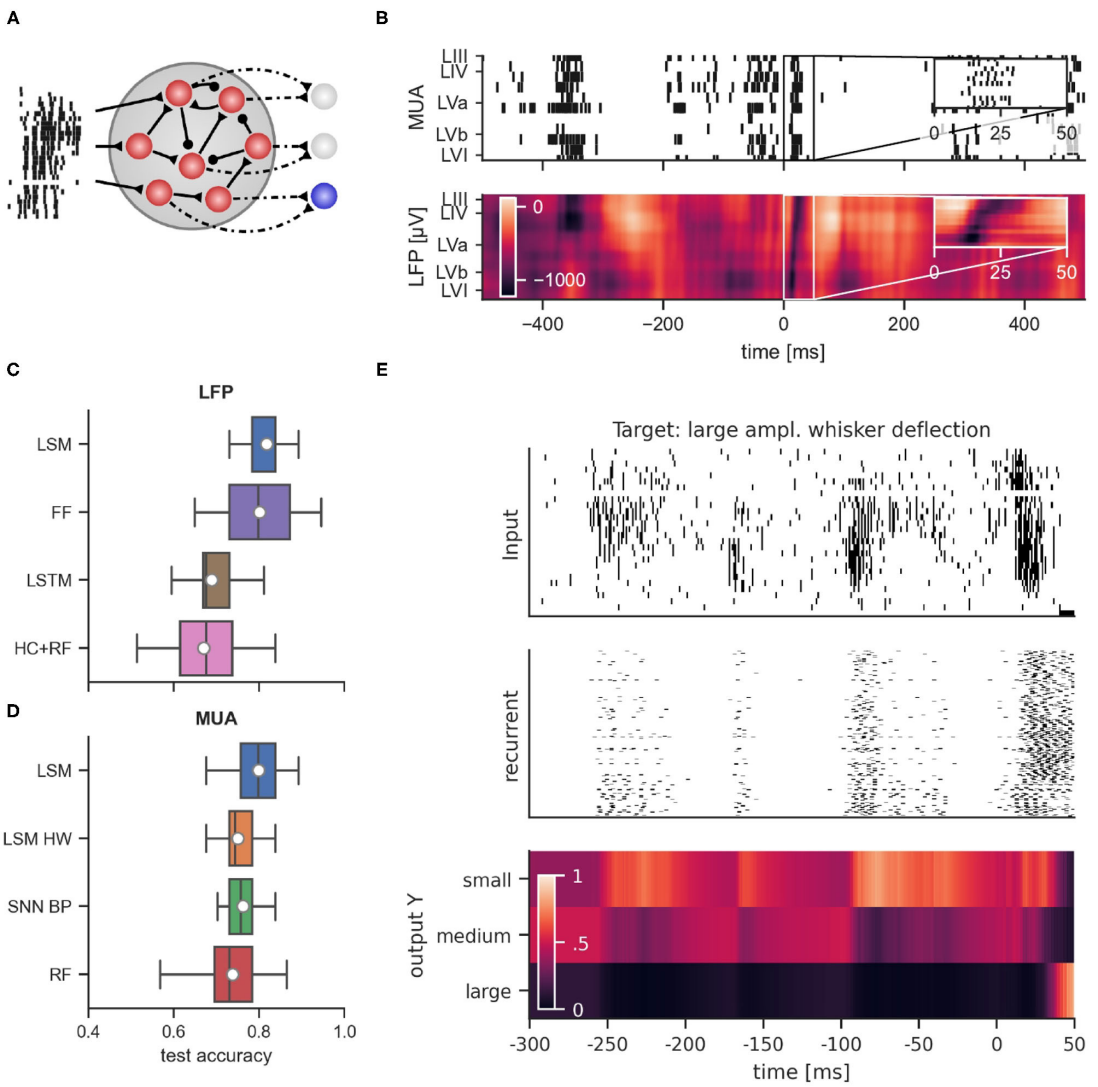
**(A)** General connectivity scheme of the recurrent spiking neural networks used in simulation. **(B)** Example of multi-unit activity (top) and the corresponding LFP trace (bottom) in response to a large whisker stimulation at time 0. Performance comparison of classifiers using LFP data from layer IV **(C)** and MUA data **(D)**. LSM, Liquid state machine; FF, feed-forward neural network; LSTM, long short-term memory network; HC+RF, handcrafted features with random forest classifier; LSM HW, liquid state machine with hardware constraints; SNN BP, recurrent spiking neural network trained with backpropagation through time; RF, random forest. **(E)** Dynamics of a recurrent spiking network after training with BPTT when multi-unit activity was presented. From top to bottom: raster plot of MUA input, response of regular spiking neurons, and activation of readout units for large, medium and small whisker deflection amplitudes. Shown is multi-unit activity and network response relative to stimulus onset starting at 300 ms before stimulus onset. Note the prominent spontaneous events which induce significant but temporally decaying responses in the SNN.

Due to its power efficiency, neuromorphic hardware that implements SNNs is a promising candidate for the on-line processing of brain-derived signals, in particular if self-contained wearable or implantable setups are considered. In order to evaluate whether SNNs can rival traditional approaches also in terms of classification accuracy, we trained SNN models on the data set described above. The goal of the training was to classify whether large, medium, or small amplitude whisker deflections were applied based on the recorded signals. Since on-line supervised training of SNNs in neuromorphic hardware is challenging, we first considered the liquid state machine (LSM) approach (Maass et al., 2002). In this approach, a recurrent SNN (the “liquid”) with fixed connectivity and weights responds to incoming stimuli according to its internal dynamics. The high-dimensional dynamics of the liquid acts on the input signal as a highly nonlinear fading memory filter and projects the signal to a high-dimensional space. By adapting the weights of connections from the liquid to a set of non-spiking readout neurons, the system can then be trained for a specific desired functionality (see Figure 4A for the network structure). One advantage of this approach is that only the weights to the readout neurons are adapted, which significantly simplifies the training procedure that has to be implemented in hardware. From the experimental setup, two types of signals were available: first, the LFP signal which is an analog low-dimensional signal, and second, the MUA which is high-dimensional and event-based. Figure 4B shows a typical MUA (top) and the corresponding LFP (bottom) trace recorded across all cortical layers over a 1 s time-window with a whisker stimulation applied in the middle of the window. An evoked response spanning all layers appears slightly after the stimulus delivery in both MUA and LFP plots. For all classifiers, we used 80% of the data for training and the remaining 20% of the data for testing. In the following sections, we evaluate the classification of stimulus intensity based on each of these two signals separately.

#### 2.3.1. Stimulus Intensity Classification Based on Local Field Potentials

We used a LSM consisting of leaky integrate-and-fire neurons to classify the stimulus intensity into three categories corresponding to large, medium, and small amplitude whisker deflections. In principle, the analog LFP signal can be injected as currents into spiking neurons of the liquid in order to provide input to the network. However, spike-based neuromorphic hardware usually only accepts spiking inputs in the form of an address event representation (Davies et al., 2018; Moradi et al., 2018). Therefore we encoded the analog LFP signal (recorded from layer IV, see Figure 1B) as spike events and presented these events to the SNN as input. To that end, we adopted a simple threshold-crossing encoding approach: The input to the network consisted of 2*N*_thresh_ spiking channels, with *N*_thresh_ = 50. Channels *i* = 1, …, *N*_thresh_ emitted a spike when the analog signal rose above threshold ϑ_*i*_ in a rising phase of the LFP. Channels *i* = *N*_thresh_ + 1, …, 2*N*_thresh_ had the same thresholds, but spiked when the signal fell below threshold ϑ_*i*_ in a falling phase of the LFP. The thresholds were uniformly spaced over the signal range. The number of thresholds for the encoding was determined *via* cross-validation. In fact, while a finer granularity of the encoding (i.e., more thresholds) increases the information available to the SNN, it also increases the input dimensionality which can negatively impact network generalization, see Supplementary Figure 1. Noteworthy, compared to conversion approaches based on inhomogeneous Poisson point processes, this approach produces a much sparser representation of the signal. The spike-encoded LFP signal was fed into a subset of the LSM neuron population. This setup achieved a mean test accuracy of 81.6 ± 4.1% (mean ± standard deviation). The average network dynamics after stimulation onset for the three different stimulation amplitudes is shown in Supplementary Figure 2.

While LSMs can solve complex tasks with randomly chosen synaptic connections and trained linear readout units, ideas from deep learning may further boost the performance of recurrent spiking neural networks. Backpropagation through time (BPTT) is a powerful optimization method that can adapt network weights while instilling desirable properties such as network sparsity or low firing activity into a network. Bellec et al. (2018b) have shown that recurrent spiking neural networks can successfully be trained with BPTT to achieve performances on temporal computing tasks that approach those of state-of-the-art artificial recurrent neural networks. We therefore wondered whether full training of SNNs with BPTT can improve their stimulus classification performance. To this end, we trained SNNs with BPTT in the same setup, which achieved a mean test accuracy of 69.8 ± 8.9%. This shows that the simple LSM approach outperforms the much more complex BPTT training algorithm which adapts all network weights. We suspect that the inferior performance of BPTT is due to the small training set size, which can lead to severe overfitting. This occurred despite the use of weight regularization during training. We found a similar performance gap when classification was based on multi-unit activity, see further discussion in the following subsection.

We compared the SNN performance to traditional non-spiking machine learning methods such as feed-forward neural networks, long short-term memory networks (LSTMs), and random forests. The results are summarized in Figure 4C. We first trained feed-forward neural networks with one hidden layer which received the values of the LFP during the first 50 ms after stimulus onset as input, where the LFP signal was sampled at 1 kHz. Hence, for each training example, the networks received a 50-dimensional input vector. This setup achieved a classification performance comparably to LSMs (FF, 80.1 ± 8.6%). The feed-forward network exhibited a higher variance compared to LSMs and required a more complex training method (L-BFGS) to achieve this result. Next, we trained LSTMs which received the LFP signal in the same input window, but in sequential order through a single input neuron. The performance of LSTMs was considerably worse (LSTM, 68.9 ± 6.5%). Previous studies (Temereanca and Simons, 2003; Mahmud et al., 2016; Wang et al., 2018) relied on engineered features that were extracted from the LFP signal. We wondered whether such hand-crafted features could improve classification performance. We therefore extracted features as proposed in these studies from the LFP signal and trained a random forest classifier (Breiman, 2001) on these features (see Section 4.6.2 for the list of extracted features). However, we found that this approach performed worse than our LSM trained on the spike-based input or the neural network trained directly on the LFP (HC-RF: 66.4 ± 6.6%).

In summary, we found that LSMs performed slightly better than traditional machine learning approaches and outperformed LSTMs. Their performance was superior to SNNs trained with the more complex BPTT training algorithm. We also found that for LSMs, spiking input is viable. A simple threshold encoding of the LFP signal is sufficient. Note that while the threshold encoding can also be seen as a kind of feature extraction from the LFP signal, it is qualitatively different from the handcrafted features we considered above: While the handcrafted features have been designed based on the knowledge about LFP signal characteristics, the threshold encoding is a more general approach that can be applied to any continuous signal without domain-specific knowledge.

#### 2.3.2. Stimulus Intensity Classification Based on Multi-Unit Activity

In addition to LFPs, MUA in the frequency range between 300 and 3,000 Hz was considered. Spikes were extracted from MUAs by a threshold approach (see Section 4) and no spike-sorting was applied. Since neural responses of single cortical neurons are known to be very noisy and the number of electrodes per layer was very limited in our data set, the signal-to-noise ratio of spike trains of individual layers was low. Therefore, for our spiking classifiers, thresholded spike events from all 27 electrodes were directly provided as inputs to the networks. MUA data from rat barrel cortex includes channels that exhibit a strong response to whisker stimulation (see Figure 4B for a comparison between the MUA and LFP response to the same stimulus).

**Figure 5:**
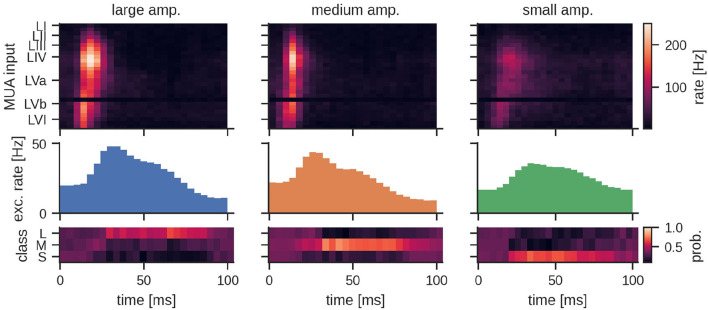
Peristimulus time histogram (PSTH) for MUA inputs and the excitatory population activity of the LSM for the three different stimulation amplitudes. Layers III, IV, and Va exhibit a particularly high firing rate depending on the amplitude of the stimulation. The bottom row shows the mean predicted class probability (Large, Medium, and Small stimulation amplitude) for the corresponding examples in the test set. See Section 4 for details.

The general network architecture and setup for classifying MUA closely followed the LSM approach described above that used (encoded) LFP signals. While the general connectivity scheme remains the same, the main difference lies in the way the input is provided to the LSM. By nature, recurrent SNNs are well suited for processing bio-signals in form of MUA. Hence, the MUA activity was directly used as input to the LSM. Using this setup, we achieved a mean test accuracy of 79.3 ± 6.5%, which is comparable to the classification performance with LFP input. The average network dynamics after stimulation onset for the three different stimulation amplitudes is reported in Figure 5.

As with the classification based on LFPs, we asked whether full SNN training with BPTT could improve performance. To this end, we trained SNNs on MUA inputs similarly to the setup with LFP input. Figure 4E reports an example of the network dynamics evolving after MUA delivery to the SNN. We found that SNNs trained with BPTT did not show improved classification accuracy compared to LSMs. In this scenario, we achieved a classification performance of 76.2 ± 4.5% on the test set. In order to see whether this inferior performance was caused by overfitting on the rather small training set, we trained both LSMs and SNNs on reduced training set sizes (see Supplementary Figure 3). Indeed, we found that the test performance of SNNs trained with BPTT declined more rapidly when training set size was decreased, suggesting that the performance gap is caused by the limited number of training examples.

For comparison with a standard non-spiking machine learning technique, we trained a random forest (RF) classifier using decision trees. Since MUA activity cannot directly be used as input to RFs, we converted MUA data into real-valued vectors in the following way: The spike trains were low-pass filtered with an exponential kernel and the filter output at classification time was used as input for the classifier. The mean test accuracy with RFs was 73.8 ± 7.6% (see Figure 4D for a visual comparison).

The classification experiments described above were performed with an ideal LSM. Ultimately, the goal is to perform classification on low-power neuromorphic hardware which is typically constrained and exhibits hardware mismatch. In order to evaluate whether the approach is viable in this scenario, we performed additional experiments with such constraints. As a test system we chose the DYNAP-SE neuromorphic processor, a mixed-signal chip with analog neurons and a very favorable energy budget (Moradi et al., 2018). We simulated properties of the DYNAP-SE neuromorphic processor such as hardware mismatch, limited fan-in and quantized weights (see Section 4 for details). With those limitations implemented in simulation, we found that the classification accuracy with an equivalent LSM setup decreased slightly to 75.1 ± 5.1% test accuracy.

#### 2.3.3. Influence of Global Network State on Classification

Certain types of anesthesia such as urethane and ketamine/xylazine have been observed to induce slow and synchronized sleep-like oscillations (Steriade et al., 1993; Sanchez-Vives and McCormick, 2000; Destexhe et al., 2007). It is known that during phases dominated by such slow oscillations, cortical neurons synchronously switch between strongly hyperpolarized (DOWN state) and depolarized (UP state) membrane potential regimes. Petersen et al. (2003) suggest that most of the trial-to-trial variability, leading, e.g., to reduced amplitude, and duration of the stimulus-evoked response can be attributed to coincidence with different phases of spontaneous activity in barrel cortex. Figure 1B shows the mean and single-trial evoked responses to whisker stimulation corresponding to large amplitude whisker deflection during UP/DOWN states. It is evident that the signal exhibits more variability during UP states than during DOWN states. Supplementary Figure 4 shows the mean LFP signal for large, medium and small amplitude stimulation intensities across all layers grouped by global network state at the time of stimulus delivery.

In order to investigate the effect of UP and DOWN states on classification accuracy, we reconsidered whisker-stimulation classification. To this end, we trained classifiers on both UP and DOWN state data and tested on UP state data and DOWN state data separately; we split the test data set into subsets consisting only of UP or DOWN states, respectively (see Section 4 for details on detecting UP and DOWN states). While the experimental design ensured an overall balanced class distribution, the distribution of UP and DOWN states during stimulus delivery could not be controlled. Overall, 58.3% of stimuli were delivered during an UP state and 41.7% during a DOWN state.

We first investigated the effect of UP and DOWN states on classifiers trained on MUA data. We evaluated whether standard machine learning approaches would be sensitive to the dynamical state of the biological network using a random forest classifier as an example. The random forest classifier trained on the combined UP+DOWN state data achieved a mean accuracy of 72.7 ± 10.9% on the UP state test set and 73.8 ± 9.3% on the DOWN state test set. A LSM trained on MUA data achieved a mean accuracy of 78.4 ± 6.6% on the UP state test set and 80.6 ± 9.6% on the DOWN state test set. This indicates that the MUA-based classifiers are not susceptible to the influence of the network state.

Next, we investigated the effect of UP and DOWN states on classifiers trained on LFP data. Following the same procedure as described above, we first evaluated random forests using the hand-crafted features extracted from the LFP. The random forest achieved a mean accuracy of 59.1 ± 9.1% on the UP state test set and 76.8 ± 10.0% on the DOWN state test set. A LSM trained on threshold-crossing encoded LFP data achieved a mean test accuracy of 81.4 ± 4.5% on the UP state test set and 81.7 ± 7.3% on the DOWN state test set. This indicates that classifiers trained on hand-crafted features are more affected by the global network state.

#### 2.3.4. Influence of Electrode Depth on Classification Accuracy

**Figure 6:**
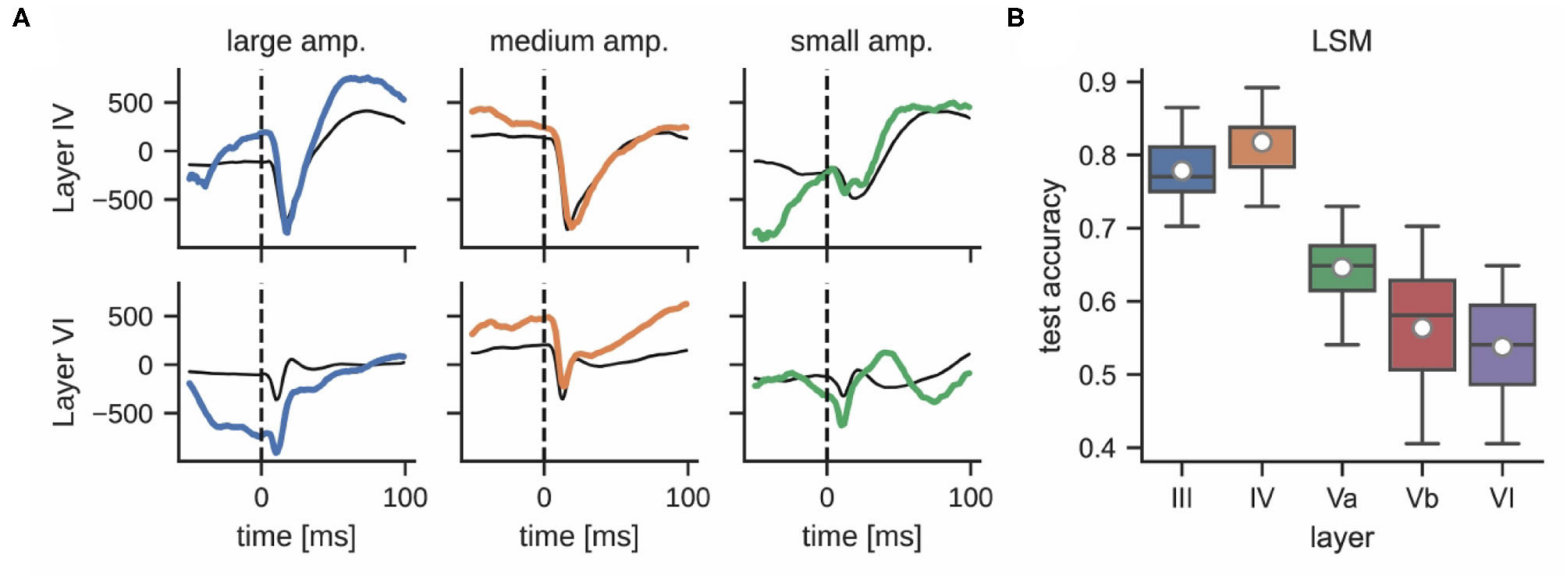
**(A)** LFPs in layer IV (top) and layer VI (bottom) after delivering a single stimulus for each whisker deflection amplitude (same color corresponds to same stimulation trial, units in μ*V*). The response of these layers to the same stimulation differs significantly; the thin black line shows the mean signal per layer and stimulation intensity. **(B)** Classification accuracy of a liquid state machine grouped by layer.

While the interaction and connectivity structure between cortical columns and layers has been studied in great detail (Feldmeyer et al., 2013), the decodability of neuronal responses after whisker stimulation with respect to the function of individual cortical layers remains unclear. Figure 6A shows that LFPs differ significantly in different layers during a single stimulation trial. For example, the responses in layer III and layer IV are more pronounced than in layer VI; see Supplementary Figure 4 for a comparison of mean LFP signals across all layers. This observation motivated an analysis that compares the classification accuracies of LSMs using LFP data from different cortical layers. Layers III and IV performed best (77.8 ± 5.1% and 81.7 ± 4.1%, respectively) whereas layers Va, Vb, and VI exhibited a decrease in performance (64.6 ± 7.0%, 56.4 ± 8.4%, and 53.7 ± 6.5%, respectively); see Figure 6B. This may be due to the fact that the ventroposterior medial nucleus mainly projects thalamic inputs onto layer IV of rat barrel cortex (Lübke and Feldmeyer, 2007), designating it as one of the most informative recording sites for our experiments. We did not perform the same analysis for multi-unit activity as the small number of electrodes per layer would result in very low classification accuracies.

## 3. Discussion

One important step toward the use of spike-based neuromorphic hardware in neuroprosthetic applications is to demonstrate that SNNs are capable of extracting useful information from a brain activity readout. To this end, we relied on the whisker somatosensory system in this work, a well defined cortical circuit whose inputs can be tightly controlled. Specifically, the unique somatotopic organization of the somatosensory barrel cortex is such that tactile receptors of a single whisker map to a single barrel column of the somatosensory cortex (Diamond et al., 2008). Thus, whisker displacements evoke in the corresponding cortical column responses that can be read out using a penetrating array of microelectrodes (Figure 1A). Moreover, single whisker movements can be controlled in the anesthetized animal with high accuracy, e.g., in our case, through a piezoelectric closed-loop system, thereby enabling a fine tuning of the sensory input. Leveraging this experimental setting, we investigated whether whisker displacements of different amplitudes could be classified by an SNN from the electrophysiological readout within the barrel column. We explored both spikes and LFP responses, as both were good candidates to carry relevant information about the sensory input for the SNN to perform the classification task. Spikes are known to convey information about whisker deflection, especially through their timing (Panzeri and Diamond, 2010). Information carried by LFPs is debated and more challenging to relate to underlying neural activity owing to the lumped nature of the signal. However, it is recognized that they do carry information (Quiroga and Panzeri, 2009; Einevoll et al., 2013) such that, for this reason, they are also used in clinical practice for neuromodulation and neurosurgery purposes (Tekriwal et al., 2019). An additional important aspect is the reliability of the classification performance taking into account that the cortical evoked responses are deeply influenced by the underlying spontaneous brain activity (Panzeri et al., 2016). Under our anesthesia conditions (urethane) the resting state is dominated by the low frequency (approximately 1 Hz) alternation of UP and DOWN states, which are signatures of high and low cortical network activity, respectively. It was therefore insightful to investigate the evoked sensory responses and SNN classification performances under these two “polarities” of cortical activity.

Our results indicate that classification of whisker stimulation intensity works well with a liquid state machine approach that is suitable for hardware implementation. Interestingly, this rather simple approach outperformed more sophisticated methods such as LSTMs or SNNs trained with BPTT. We hypothesize that due to their increased complexity, these latter approaches tend to overfit the training data. We note that these results were obtained using an extensive parameter sweep including regularization methods to avoid overfitting (see Section 4). Hence, inferiority of these approaches is not likely to be a result of insufficient parameter tuning. One possibility to improve classification results would be to increase the training set size. However, it is expected that neuroprosthetic devices need to be tuned individually per subject, which may preclude the generation of large data sets. Another finding of our study is that SNNs do not necessitate elaborate data preprocessing. We found that using either multi-unit activity directly or a simple threshold encoding of LFPs as input to the LSM leads to good performance. This is in contrast to the standard approach where specifically engineered features are extracted from the raw signal. Interestingly, we found that random forests—a state-of-the art classifier—applied on features that were previously proposed (Temereanca and Simons, 2003; Mahmud et al., 2016; Wang et al., 2018) performed clearly worse than a LSM trained directly on MUA or LFP input. Although our statistical analysis showed that standard features—in particular those extracted from the LFP—do carry information about the stimulus, this indicates that SNNs can utilize information in the signal that is hidden when such features are considered. It is possible to perform the threshold encoding of LFPs in an on-line fashion. For example, a microcontroller can encode the LFPs at a high frequency since this method is computationally inexpensive and requires minimal memory. Other solutions using special purpose hardware that encodes the events asynchronously—very similar to spiking neurons—could be implemented as well. From the perspective of brain-computer interfacing, LFP signals are preferable as they are more robust and reliable over time with respect to MUA (and spikes) in chronic conditions. Thus, our results in the somatosensory barrel cortex would favor the use of LFP in future applications. However, this may not be true for other brain areas. Sensory areas are at an early stage in the brain processing chain and the sensory stimulus is probably mapped with a high degree of redundancy across the neuronal population. This ensures that LFP (typically reflecting population activity) are effective. In other regions, such as in high-order processing (i.e., associative) areas, the representation of complex features may generate neuronal specialization. In this case, single or a few neurons, detected as spikes or MUA, may turn out to be more informative.

Our proposed LSM approach has the advantage that only the weights to the readout neurons are adapted, which significantly simplifies the training procedure. While network dynamics of a LSM can easily be implemented with available neuromorphic hardware, there is currently no integrated solution to train readout units using support vector machine training or other methods such as linear least squares. These training steps could be performed efficiently on a microcontroller. Note that in all our experiments, we were using a linear support vector machine as the readout, which can be trained efficiently using a simple gradient descent scheme on the output weights using the hinge loss. Alternatively, a softmax output layer with a cross-entropy loss could be used as well. In both cases, training can be performed in an on-line fashion. Alternatively, there exist a number of methods to train spiking readouts such as ReSuMe (Ponulak and Kasiński, 2010), the tempotron learning rule (Gütig and Sompolinsky, 2006), or standard surrogate gradient training (Bellec et al., 2018b). However, in this case, specific on-chip learning capabilities have to be considered when the neuromorphic hardware is designed. The neuron parameters in our simulations have been chosen such that the network can be ported to the DYNAP-SE processor (Moradi et al., 2018). We found that taking further hardware constraints into account leads to only a slight decrease of classification performance which is still on par with traditional machine learning approaches. In summary, our results indicate that low-power signal processing with spike-based neuromorphic hardware is a viable alternative to traditional approaches based on standard computers, leading to a novel paradigm for neuroprosthetics where spiking neurons in silicon read out and control activity of biological spiking neural networks.

## 4. Methods

### 4.1. Ethics Statement

All the experimental procedures were approved by the Animal Care Committee of the University of Padova (O.P.B.A.) and the Italian Ministry of Health (authorization number 522/2018-PR).

### 4.2. Surgical Procedures

Wistar rats were maintained in the animal research facility of the Department of Biomedical Sciences, University of Padova. Young adult rats aged 35–45 days (P35–P45; 175–230 g) were anesthetized with an intra-peritoneal induction mixture of Tiletamine-Xylazine (2 mg and 1.4 g/100 g body weight, respectively), followed every hour by additional doses (0.5 mg and 0.5 g/100 g body weight). The absence of eye and hind-limb reflexes and whiskers' spontaneous movements indicated a good anesthesia level. The rat was laid on a heating pad which maintained the body temperature at 37 °C through a rectal probe, and fixed on a stereotaxic apparatus by teeth and ear bars. A window on the exposed skull was drilled over the right somatosensory barrel cortex S1 at stereotaxic coordinates −1 to −4 AP, +4 to +8 ML referred to bregma (Swanson, 2004). The brain was constantly bathed in Krebs' solution (in mM: NaCl 120, KCl 1.99, NaHCO_3_ 25.56, KH_2_PO_4_ 136.09, CaCl_2_ 2, MgSO_4_ 1.2, glucose 11).

### 4.3. Whisker Stimulation and Extracellular MUAs and LFPs Recordings

Contralateral whiskers were trimmed at 10 mm from the mystacial pad. Whiskers were individually inserted into a cannula glued to a piezoelectric bender with integrated strain gauges [P-871.122; Physik Instrumente (PI) GmbH & Co. KG], driven by a home-made closed-loop control system and connected to a dedicated amplifier [E-650.00 LVPZT-amplifier; Physik Instrumente (PI) GmbH & Co. KG]. A 5 ms voltage pulse stimulus with 100 μs rise/fall time and 2.8 V amplitude (upper limit, which corresponds to final 60 V) was applied to the bender through a waveform generator (Agilent 33250A 80 MHz, Agilent Technologies Inc.). The whisker providing the maximum amplitude of evoked LFP (“principal whisker”) was selected for the recording session, which consisted of random and/or sequence pulse stimulations with 5 ms duration and variable amplitude provided with 10 s interval between subsequent stimuli. Each stimulation trial in a recording session was preceded and followed by 5 min of rest. Intracortical signals were recorded by a linear 32-electrodes-silicon probe (E32+R-65-S1-L6-NT; 65 μm spaced Iridium Oxide (IrOx) microelectrodes with a mean impedance of 0.28 MΩ in Krebs' solution at 1 KHz; ATLAS Neuroengineering, Belgium). For signal amplification and acquisition, the probe was connected to a 32-channels head stage (RHD2000, Intan Technologies) and to the Open Ephys acquisition system (OEps Tech, Portugal), and then data stored in a PC. Raw traces were bandpass filtered offline at 0.1–300 and 300–3,000 Hz for MUAs and LFPs, respectively. An I/O board connected to the Open Ephys system with a HDMI cable was used to synchronize the piezoelectric stimulation with signal acquisition. The probe was inserted in the somatosensory cortex, through a slit in the meninges, with a PatchStar micromanipulator (Scientifica Ltd) following a direction orthogonal to the brain surface at coordinates −2.5 AP, +6 ML. The depth was set at 0 μm when the electrode proximal to the chip tip touched the cortical surface. The array was covering all the six cortical layers (from 0 to −1,800 μm). An Ag/AgCl electrode bathed in Krebs' solution was used as reference. Raw signals were visualized, recorded and digitalized at 25 kHz through the Graphic User Interface software supplied with the Open Ephys acquisition system.

### 4.4. Extraction of Multi Unit Activity

Spikes were detected using the WaveClus algorithm (Quiroga et al., 2004), an unsupervised and fast method which has been tested and validated on both *in vitro* and *in vivo* electrophysiological data and for real-time applications. In particular, spike detection was performed by setting an automatic amplitude thresholding after the raw recordings were filtered between 300 and 3,000 Hz using a digital elliptic filter. The threshold (Thr) was automatically set to:


$$\text{Thr}=3\sigma_{n},$$

where σ_*n*_ is an estimate of the standard deviation of the background noise. Indeed, taking the standard deviation of the entire signal (i.e., including spikes) could lead to very high threshold values, especially in cases with high firing rates and large spike amplitudes, with a consequent increase of false negative detection. The adopted *spike detection* method allowed to identify both isolated spikes and the so-called Multi-Unit Activity (*MUA*) events, i.e., packed spiking events originated by the quasi-simultaneous activity of a population of neurons located nearby each electrode. Specifically, a (*MUA*) was defined as a set of spikes occurring less than 20 ms one to each other.

### 4.5. Distinction Between Stimuli Delivered During a DOWN State and an UP State

One of the objective of this work consisted in understanding how ongoing basal activity affects evoked responses, making it necessary to distinguish between two different dynamical states, i.e., the DOWN and the UP state, indicating, respectively, a system being in a quiescent and in a dynamically active period.

To this aim, the Instantaneous Firing Rate (IFR) was computed for each electrode as the average number of spikes present within sliding windows of arbitrary width. For this application, a moving window of 10 ms and a sliding temporal step of 1 ms were adopted in order to depict the fast dynamics characterizing MUA events.

Successively, the cumulative IFR profile was obtained by summing all electrodes' IFR, in order to extract the dynamics at network level. The adoption of this parameter allowed us to distinguish between the two dynamical states by setting the global average of the cumulative IFR profile as threshold. So, the detected network events, identifying UP states, are the result of the superimposition of MUA events observed across all electrodes. Finally, in order to distinguish between stimuli occurring during a DOWN or during an UP state period, a fixed investigation window of 50 ms was set before each stimulus' occurrence. Specifically, a stimulus was classified as occurring during an UP state if an UP state was identified within the investigation window.

### 4.6. Details to: Statistical Analysis of Features Extracted From Evoked Responses

#### 4.6.1. Feature Extraction From Stimulus-Evoked Spiking Dynamics

Depending on whether the stimulus is delivered during a DOWN or during an UP state, the system can exhibit different dynamical behaviors which have to be investigated in the perspectives of developing an automatic activity pattern recognition approach. To this aim, three characteristic spiking features were extracted from the evoked responses at the level of single cortical layer: *MUA latency, MUA duration*, and *MUA firing rate*. The MUA evoked within each investigated layer was computed by summing all spikes detected at the level of single electrodes belonging to each layer.

**Figure 7:**
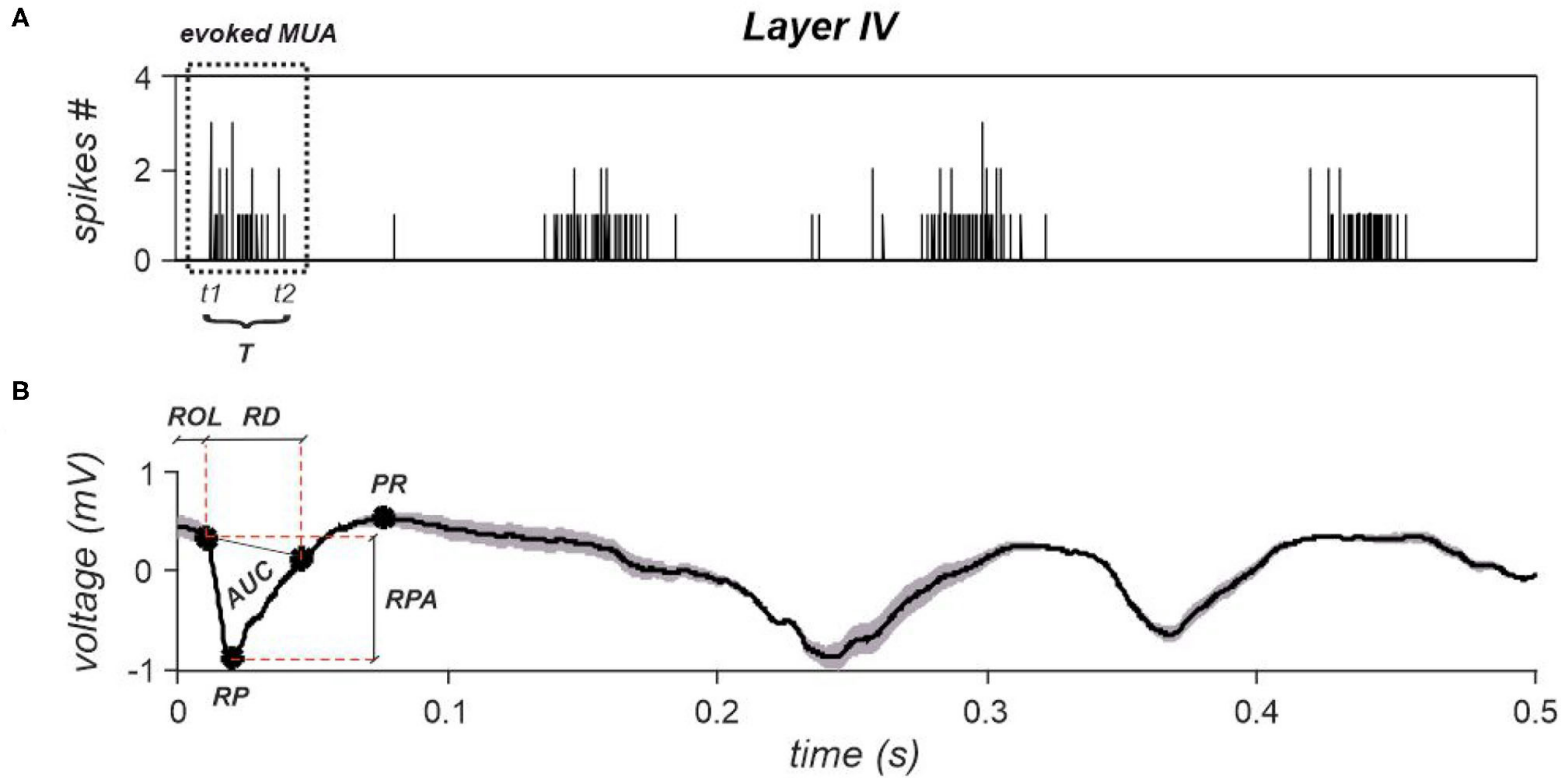
**(A)** A cumulative spike train obtained by summing spikes detected from all electrodes belonging to layer IV. Time instant zero indicates stimulation's occurrence. The evoked *MUA* is highlighted by a dotted rectangle, whose start and end are indicated as *t1* and *t2*. The duration, *T* is also reported. **(B)** A representative evoked LFP obtained by averaging all evoked responses observed within layer IV. The corresponding extracted features are reported, i.e., *ROL, RPA*, and *PR*. *AUC* and *RD* are the parameters necessary to compute the last investigated LFPs' feature, i.e., *tLFP*.

Specifically, the evoked *MUA* latency was computed as the temporal instant at which the first spike occurred after each stimulus (denoted as *t1*—Figure 7A). The evoked *MUA* end, indicated as *t2*, instead, corresponds to the time instant at which the last spike of the evoked MUA occurred. So, its duration *T* was computed as the difference between *t2* and *t1* (Figure 7A). Finally, the evoked *MUA firing rate* was computed as the ratio between the number of spikes belonging to the MUAs evoked within each layer and its duration *T*.

#### 4.6.2. Feature Extraction From Stimulus-Evoked LFPs

LFP signals were extracted by bandpass filtering raw traces between 0.1 and 300 Hz using a digital elliptic filter. Subsequently, in order to lighten the computational cost, LFPs were downsampled in order to obtain a final sampling frequency of 2,500 Hz. Subsequently, an investigation window of 3 s was selected, starting from stimulus occurrence, for all delivered stimuli in order to isolate and characterize the evoked responses.

Four features were extracted per each stimulation intensity and by discriminating between stimuli delivered during a DOWN state and stimuli delivered during an UP state. Specifically, the examined features are the following: *i)* Response Peak Amplitude (*RPA*), *ii)* Positive Rebound (*PR*), *iii)* Response Onset Latency (*ROL*); and *iv)* time-normalized LFP (*tLFP*). Representative features, extracted from layer IV, are shown in Figure 7B.

To this aim, LFPs were averaged across all electrodes belonging to each layer in order to remove noisy components that could compromise features' extraction. Furthermore, the average signals' first derivative was computed within the first 20 ms from stimulus' occurrence, and the (*ROL*) was extracted by computing the instant at which the absolute value of the derivative overcomes a specific threshold, which was set as follows:


$$\text{th}{\text{r}}_{\text{latency}}=\text{mea}{\text{n}}_{\text{ba}{\text{s}}^{\prime }}+3\sigma_{\text{ba}{\text{s}}^{\prime }}$$

where bas′ indicates the first derivative of the baseline activity extracted from a 10 s window preceding each stimulus.

The *RPA* was extracted from the portion of signal starting at the onset latency instant till the following 50 ms, by detecting the response peak (*RP*). Then, the *RPA* was computed as the sum in voltage of the *RP* and the response's amplitude at the previously computed onset latency. Due to the slower dynamic characterizing the second part of the evoked LFP response, the *PR*, where present, was computed as the maximum positive value in voltage detected within the 100 ms following the main response peak, as described in Wang et al. (2018).

Finally, *tLFP* was computed as follows:


$$\text{tLFP}=\frac{\text{AUC}}{\text{RD}}$$

where AUC and RD are, respectively, the area under the main response peak and the response duration, as shown in Figure 7B for layer IV.

#### 4.6.3. Statistical Analysis

The performed statistical analysis aimed at comparing, per each stimulation intensity, the investigated features extracted from responses following both a DOWN state and an UP state. To this aim, the non-parametric Wilcoxon rank-sum test was performed.

A multiple comparison among the three different stimulation intensities (i.e., 2.8, 2, 1.6 V) was also performed by adopting the non-parametric Kruskal–Wallis test and by applying the Dunn's test (a non-parametric pairwise multiple-comparison procedure) when the Kruskal–Wallis test was rejected.

### 4.7. Stimulation Classification

#### 4.7.1. Liquid State Machines

Liquid state machines (Maass et al., 2002) have been shown to work well on the current generation of neuromorphic processors despite hardware limitations: The concept of recurrent spiking neural networks with static and randomly drawn connection weights lends itself to implementations on neuromorphic hardware. Liquid state machines (LSMs) in form of such circuits integrate temporal information so that linear readouts trained on the network activity can solve complex computational tasks. We used a recurrent spiking neural network with leaky integrate-and-fire (LIF) neurons with exponential shaped postsynaptic currents and static synapses. Neuron parameters have been adapted from Maass et al. (2002) with some modifications to the connectivity scheme. Simulations have been carried out in NEST 2.18 (Gewaltig and Diesmann, 2007). The neural circuit was composed of excitatory neurons and inhibitory neurons. Each neuron received input from a fixed number of excitatory and inhibitory neurons chosen at random. Synaptic weights depended on whether the pre- and postsynaptic neuron were excitatory (E) or inhibitory (I). Network weights have been scaled to produce a mean firing rate below 50 Hz while maintaining good classification accuracy. Each input was projected onto a subset the excitatory population. The spike trains of the excitatory population were low-pass filtered with an exponential kernel. This trace of integrated information was then fed into a linear support vector machine. Finally, the linear classifier was trained to distinguish different whisker stimulation intensities.

#### 4.7.2. SNN Trained With BPTT

The main characteristic of liquid state machines is that only the synaptic weights to the readout neurons are adapted while the other weights are fixed and chosen at random. Conversely, Bellec et al. (2018b) have shown that a variant of backpropagation through time can be used to train recurrent networks of leaky integrate-and-fire neurons end-to-end. LIF neurons are simulated in discrete time with a timestep of δ*t* = 1ms. The membrane potential *V*_*j*_(*t*) of a LIF neuron *j* evolves as follows:


$$V_{j}\left(t+\delta t\right)=\alpha_{j}V_{j}\left(t\right)+\left(1-\alpha_{j}\right)R_{m}I_{j}\left(t\right),$$

where the decay $\alpha_{j}=\exp\left(-\frac{\delta t}{\tau_{j}}\right)$ is defined using the membrane time constant τ_*j*_, *R*_*m*_ is the membrane resistance and *I*_*j*_(*t*) is the weighted sum of incoming spikes. After crossing the firing threshold *b*_*j*_, neuron *j* emits a spike $z_{j}\left(t\right)=\frac{1}{\delta t}$, the membrane potential is reset by subtracting the threshold value and *z*_*j*_(*t*) is fixed to zero in the following refractory period. Due to the non-differentiable nature of the firing event of spikikng neurons, the derivative of a spike with respect to the normalized membrane potential $v_{j}\left(t\right)=\frac{V_{j}\left(t\right)-b_{j}}{b_{j}}$ is replaced with a dampened pseudo-derivative


$$\frac{\partial z_{j}\left(t\right)}{\partial v_{j}\left(t\right)}=\gamma \max\left\{0,1-|v_{j}\left(t\right)|\right\},$$

where γ = 0.3 is a dampening factor. In all simulations, the membrane time constant τ_*j*_ = 20ms, the resistance *R*_*m*_ = 1*G*Ω and the threshold *b*_*j*_ = 0.01.

### 4.8. Details on Models and Training

Both the LFP and MUA datasets were split into training (80% of dataset) and test dataset (20% of dataset). The hyperparameters of all classifiers were cross-validated using grid search on a separate validation dataset (20% of training dataset). Due to the small overall dataset size (185 examples in total), the specific split into training and test set significantly affects the performance of a classifier. This necessitates an averaging of the performance metrics of classifiers over many independent runs with different splits. Therefore, for each classifier, we performed 20 independent evaluations where initial parameters and training/validation/test splits were randomly chosen for each evaluation. In each such evaluation, we performed a grid search over the considered hyperparameter values and used that values with the best validation accuracy to determine the test error of that run. The average test error was then computed as the mean over those errors. All models trained using backpropagation (through time) used the categorical cross entropy loss.

#### 4.8.1. Details to: Stimulus Intensity Classification Based on Local Field Potentials

All classifiers were trained using LFP data acquired in layer IV of barrel cortex. To this end, LFP signals were downsampled to 1 kHz and bandpass-filtered between 0.1 and 300 Hz. Then, the signals recorded from all electrodes in layer IV were averaged to obtain a single averaged LFP for layer IV.

*LSM:* We used a liquid state machine with 330 excitatory and 80 inhibitory leaky integrate-and-fire neurons with exponentially-shaped postsynaptic currents inspired by Kaiser et al. (2017). The LFP signal was converted into a set of 100 spike trains according to the threshold encoding approach described above which were provided as input to the LSM. The spikes of each channel were randomly projected onto four excitatory neurons using static synapses with weights drawn from a uniform distribution U(250,750) pA. The delays were drawn from a normal distribution with mean 10 ms, standard deviation 20 ms, and clipped to [3, 200] ms. Recurrent connections in the LSM exhibited short-term plasticity according to (Tsodyks and Markram, 1997; Fuhrmann et al., 2002). Excitatory weights, inhibitory weights and synaptic delays were constrained to [0, ∞), (−∞, 0] and [3, 200], respectively. See Tables 1, 2 for an overview of neuron and synapse parameters, respectively. The spike trains of the excitatory population were low-pass filtered with an exponential kernel with a time constant of 5 ms. This trace of integrated information was read out 45 ms after stimulus onset and fed into a support vector machine with a linear kernel.

**Table 1:** Parameters of leaky integrate-and-fire neuron model with exponential-shaped postsynaptic currents using the NEST simulator.

**Parameter**	**Value**	**Unit**
Resting membrane potential	0.0	mV
Capacity of the membrane	30.0	pF
Membrane time constant	30.0	ms
Exponential decay time constant of excitatory synaptic current	3.0	ms
Exponential decay time constant of inhibitory synaptic current	2.0	ms
Duration of refractory period	2.0	ms
Membrane potential	−70.0	mV
Spike threshold	15.0	mV
Reset membrane potential after a spike	13.8	mV
Constant input current	7.0	pA
Incoming excitatory connections	2	
Incoming inhibitory connections	1	

**Table 2:** Parameters of network connections.

**Parameter**	**Distribution**	**Unit**
Weight of EE connection	N(100,70)	pA
Weight of EI connection	N(500,350)	pA
Weight of IE connection	N(-400,280)	pA
Weight of II connection	N(-400,280)	pA
Weight of noise connection	N(2,1)	pA
Delay of connections	N(10,20)	ms

*An XY connection is a connection from a neuron in population type X to a neuron in population type Y, where X and Y can be E (exciatatory) or I (inhibitory). Here, N(μ,σ) denotes a normal distribution with mean μ and standard deviation σ*.

The LSM received data starting at 1 s before stimulation onset in order to simulate the effects of spontaneous activity on the dynamics of the LSM. The hyperparameters that were tuned in the above mentioned grid search were the scaling coefficients of input, excitatory, and inhibitory weights as well as the support vector machine readout regularization parameter *C*.

*Recurrent Spiking Neural Network with BPTT (SNN BP):* The recurrent spiking neural network trained with backpropagation through time was designed to match the structure of the LSM network. Unlike the LSM, the SNN used static synapses instead of dynamic synapses. The SNN was trained with threshold-crossing encoded LFP data within a 50 ms window after stimulation onset and stochastic gradient descent using over 500 epochs. The learning rate was reduced by a factor of two once training stalled for more than 10 epochs. Since the network structure based on the LSM was sparse, we employed a variant of Deep Rewiring (Bellec et al., 2018a) to dynamically rewire connections during the course of training and *L*_1_-norm regularization of the weights. Unlike Deep Rewiring, the target connectivity was set to match the connectivity scheme of the LSM instead of a fixed global connectivity level. The learning rate, batch size, rewiring temperature and *L*_1_-norm regularization coefficient were tuned using grid search as described above.

*Feed-forward neural network (FF):* We used a single-layer feed-forward neural network with 80 hidden units and ReLU nonlinearity. The input to the network was the LFP signal within a 50 ms window after stimulation onset, i.e., its input contained the LFP values for each sampled time *t*_onset_, *t*_onset_ + 1 ms, *t*_onset_ + 2 ms, …, *t*_onset_ + 49 ms where *t*_onset_ is the whisker stimulation time. Each input feature to the network was standardized to zero mean and unit standard deviation over the dataset. The network was trained using L-BFGS (Liu and Nocedal, 1989) with a learning rate of 0.001 for 50 epochs. We used early stopping in the sense that the parameters at the epoch that yielded the highest validation accuracy were used for testing. The weights were initialized from $U\left(-\sqrt{k},\sqrt{k}\right)$ where $k=\frac{1}{\text{in}\text{\_}\text{features}}$ and the biases were initialized to 0. The weights were regularized using *L*_2_ regularization with a regularization coefficient of 0.001.

*LSTM:* We used a long short-term memory recurrent neural network with 64 LSTM units. The LFP signal was standardized to zero mean and unit standard deviation over time. The network received input from one input neuron, where the LFP values in a 50 ms window after stimulus onset were provided sequentially. It was trained with stochastic gradient descent with a learning rate of 0.1 over 250 epochs, a momentum factor of 0.9 and a batch size of 32. We used early stopping as described above. The weights were initialized from $U\left(-\sqrt{k},\sqrt{k}\right)$ where $k=\frac{1}{\text{in}\text{\_}\text{features}}$ and the biases were initialized to 0.

*Random forest on hand-crafted features (HC+RF)*: Here we used a random forest with 100 individual decision tree classifiers as the classifier (Breiman, 2001), where we used hand-crafted features extracted from the LFP signal as input. These features were the response peak amplitude (RPA), positive rebound (PR), response onset latency (ROL) and time-normalized LFP (tLFP) as well as and the mean and standard deviation of the LFPs signal in considered window (50 ms after stimulus onset as above). The number of features per tree, the minimum number of samples required to split an internal node, and the criterion to measure split quality (Gini impurity or information gain) were tuned in the grid search mentioned above.

#### 4.8.2. Details to: Stimulus Intensity Classification Based on Multi-Unit Activity

*LSM:* We used a liquid state machine with 100 excitatory (E) and 25 inhibitory (I) leaky integrate-and-fire neurons as described in Section 4.8.1. The spike trains from each of the 27 input channels were projected onto four excitatory neurons using static synapses with weights drawn from U(15,45) pA. Delays were drawn from a normal distribution with mean 10 ms, standard deviation 20 ms, and clipped to [3, 200] ms. The LSM received data starting at 1 s before stimulation onset in order to simulate the effects of spontaneous activity on the dynamics of the LSM. The parameters of the liquid state machine (scaling coefficients of input, excitatory and inhibitory weights) as well as the linear support vector machine readout (regularization parameter *C*) were tuned using cross-validated grid search.

*Hardware-constrained LSM (LSM HW):* In order to test a hardware-constrained LSM, we considered the DYNAP-SE neuromorphic processor (Moradi et al., 2018) as a test bed. The LSM with hardware constraints had the same network structure as the regular LSM. However, in line with the hardware constraints, the network used static synapses with discretized weights matching the fan-in constraints of the DYNAP-SE neuromorphic processor. The DYNAP-SE processor has a fan-in limit of 64 connections per neuron. Each connection between two neurons has one of four possible types: slow or fast excitatory and slow or fast inhibitory. Each DYNAP-SE processor has four chips with four cores each containing 256 neurons; all synapses with the same type on the same core have the same weight. In order to achieve different connection strengths, multiple connections between two neurons can be established, as long as the fan-in constraint is fulfilled. In addition, there were no synaptic delays in the network. Since the DYNAP-SE processor has analog neurons, there are many sources of noise in the system. In order to simulate such parameter variations, parameters of individual neurons were varied around their defined values with a coefficient of variation of 20% clipped at two standard deviations. For example, if the membrane time constant of the neurons is set to 20 ms, each individual neuron's membrane time constant was drawn from a Gaussian distribution with 20 ms mean and standard deviation of 4 ms and clipped within the interval [12, 28] ms. The varied parameters were the membrane time constant, the threshold, the reset potential and the membrane capacitance.

*Recurrent Spiking Neural Network with BPTT (SNN BP):* The recurrent spiking neural network trained with backpropagation through time was designed to match the structure of the LSM network and trained as described in Section 4.8.1. The SNN was trained with MUA data within a 50 ms window after stimulation onset. and stochastic gradient descent over 500 epochs. The learning rate was reduced by a factor of two once training stalled for more than 10 epochs.

*Random forest (RF):* The spike trains were low-pass filtered with an exponential kernel with a time constant of 16 ms and read out 30 ms after stimulation onset. These features were then passed into a random forest with 100 decision trees. The number of features per tree, the minimum number of samples required to split an internal node and the criterion to measure split quality (Gini impurity or information gain) were tuned using grid search.

#### 4.8.3. Details to: LSM Population Response Figures

For Figure 5 and Supplementary Figure 2, the PSTH was computed for each of the MUA and threshold-encoded LFP channels as well as the excitatory population of the LSM with a bin size of 4 ms. For each stimulation amplitude, we show the mean predicted class probability for the corresponding examples in the test dataset over time. Since SVMs do not assign class membership probabilities by default, we resort to the method proposed by Wu et al. (2004) to estimate class probabilities using pairwise coupling.

## Data Availability Statement

The raw data supporting the conclusions of this article will be made available by the authors, without undue reservation.

## Ethics Statement

All the experimental procedures were approved by the Animal Care Committee of the University of Padova (O.P.B.A.) and the Italian Ministry of Health (authorization number 522/2018-PR).

## Author Contributions

RL and SV conceived the idea. HP performed the classification experiments and analyzed the results. MM and AL performed the electrophysiological recordings. MB performed the data processing, analysis, and statistics. RL, SV, HP, and MB wrote the manuscript. All authors contributed to the article and approved the submitted version.

## Funding

This work was supported by the grant SYNCH (European Commission, FET Proactive, GA N. 824162).

## Conflict of Interest

The authors declare that the research was conducted in the absence of any commercial or financial relationships that could be construed as a potential conflict of interest.

## Publisher's Note

All claims expressed in this article are solely those of the authors and do not necessarily represent those of their affiliated organizations, or those of the publisher, the editors and the reviewers. Any product that may be evaluated in this article, or claim that may be made by its manufacturer, is not guaranteed or endorsed by the publisher.
