# Supplementary material for: Classification of Whisker Deflections From Evoked Responses in the Somatosensory Barrel Cortex With Spiking Neural Networks
Source: Front Neurosci. 2022 Apr 14;16:838054. doi: 10.3389/fnins.2022.838054 (PMC9047904; doi:10.3389/fnins.2022.838054)
Supplement: Supplementary file 1 [file Data_Sheet_1.PDF]

frontiers

Supplementary Material

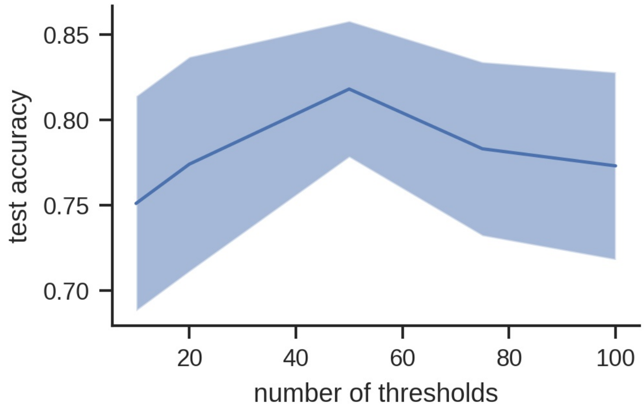

Figure S1. Mean test performance of the LSM classifier using threshold-encoded LFP signals for different numbers of thresholds N_{thresh} in the input encoding. The mean test accuracies range from $75.1\% \pm 6.3\%$ when using 10 thresholds to $81.6\% \pm 4.1\%$ when using 50 thresholds. The thresholds were uniformly spaced across the range of the signal based on the number of thresholds used.

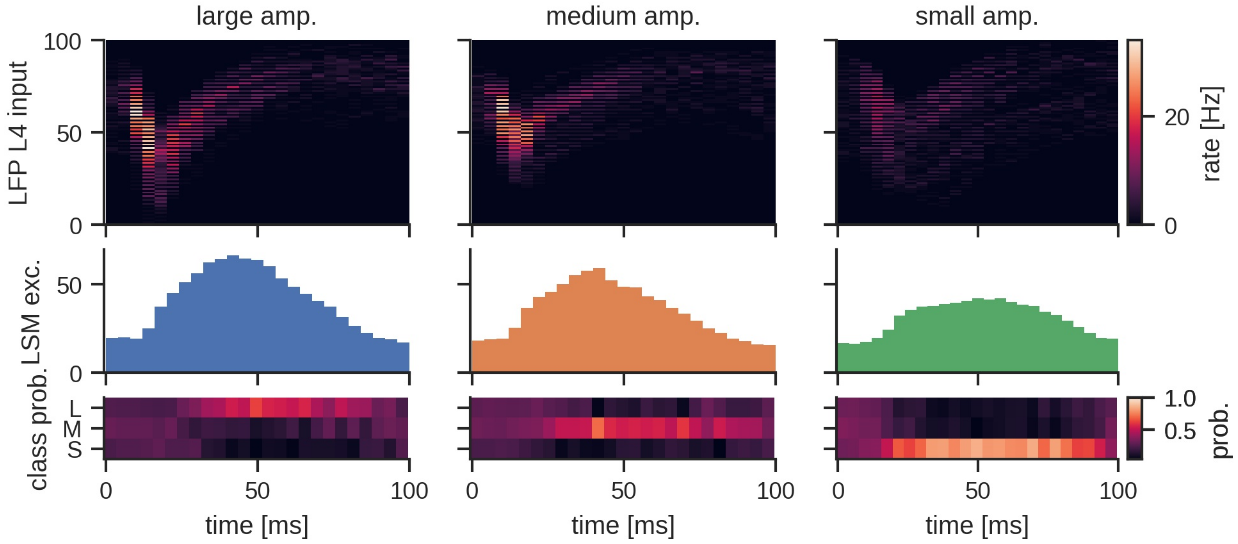

Figure S2. Peristimulus time histogram (PSTH) for threshold-encoded LFP inputs from layer IV and the excitatory population activity of the LSM for the three different stimulation amplitudes. The bottom row shows the mean class probability (Large, Medium and Small stimulation amplitude) according to the network readout over time.

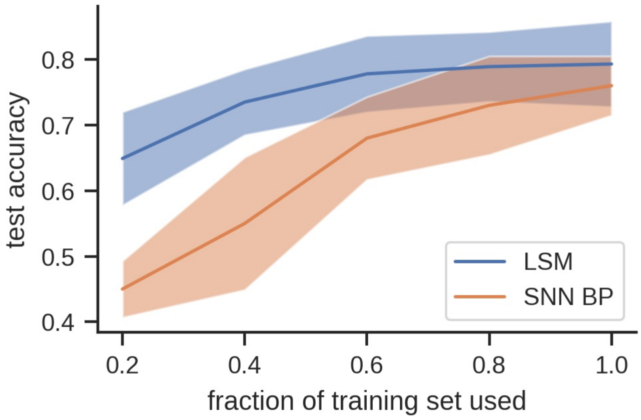

Figure S3. Mean test performance of the LSM and SNN trained with BPTT (SNN BP) using MUA inputs as considered in Section 2.3.2 for different training set sizes relative to the original training set size. The test dataset sizes remained unaffected while the training dataset sizes were reduced gradually: A randomized subset of the training dataset was sampled (while maintaining the class distribution) and used for training. For the LSM, the regularization coefficient C of the SVM readout was tuned for each fractional training dataset independently. For the SNN BP classifier, the weight regularization coefficient was tuned independently.

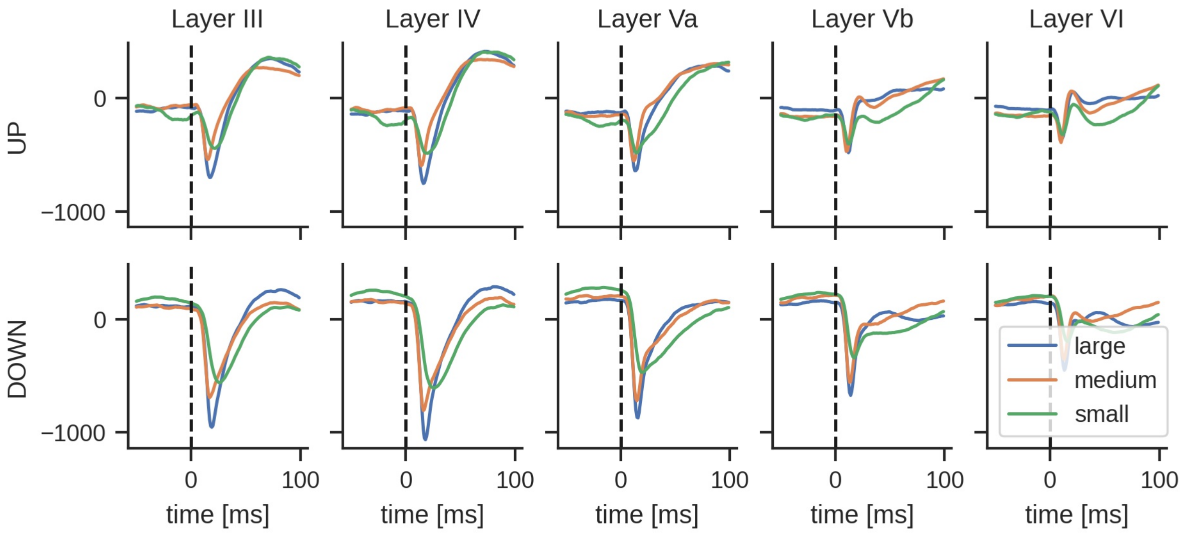

Figure S4. Comparison of mean LFP signals for large, medium and small amplitude stimulation intensities across all layers (units in μV) grouped by global network state. The dashed line indicates stimulation onset.
